# Editorial: Setting Science Free

**Published:** 2006-10-13

**Authors:** Stephen M. Milner

In the present system of scientific publishing, scientists do research, write papers, review their peers' work, edit scientific journals, and give away their copyright—all completely free of charge to the publishers. Then if they wish to learn about their work or that of their colleagues, they are expected to subscribe to expensive journals. In a world that is being transformed by high-speed computing, connecting individuals and linking institutions around the world, the sharing of scientific information in academia remains constrained by a traditional ink-on-paper system.

## LIMITATIONS OF THE CURRENT SYSTEM OF MEDICAL PUBLISHING

The current system of medical publishing is limited in a number of ways. There are long delays in the time between submission of articles and their publication, which may limit the value or practical importance to scientists had it been published sooner. The high cost of journals now puts them beyond the reach of 70% of the world, and scientific progress is hindered because researchers are isolated from their colleagues. Annual subscriptions to some scientific journals are priced as much as $20,000. The logistics of distribution further contribute to the length of time and expense from publication to receipt of the paper journal.

## HISTORY

Until recently the personal computer was an obscure experiment in university science departments and letters were written on typewriters. Today, computers and the Internet have transformed how we communicate with one another and serve as an essential link to the world. With the prevalence of computers in homes, offices, and at the bedside, such computers have become common commodities and outsell television sets. This raises the question as to why researchers still use paper journals with their inherent limitations of time, expense, and space in order to keep abreast of the latest developments in their fields? The answer lies in the history of a system that has remained stagnant for nearly half a century.

The present-day system of medical publishing dates back 400 years with the fusion of a new technology—print—and a system of public access—The Postal Service. The first academic journal in the English language, *The Philosophical Transactions of the Royal Society of London*, was created by Henry Oldenburg in 1665 as a kind of repository for intellectual property. This allowed scientists to stake their claims to original findings and provided the widest access possible to readers at that time. As specialties developed, so too did the societies, and the centerpiece of each society was the journal, which was largely responsible for funding the parent organization. Peer review was added later to give the journals legitimacy.

Throughout this era, Faustian bargains were perpetuated between the publishers and the authors. The publishers printed the journals and, in return, the authors supplied their work completely free and gave up all copyright. The societies, with a vested interest in this system, assisted the publishers by delivering healthy and compulsory subscriptions. In fairness, the system provided the very best in its time. Even today there are many faculty members who prefer their journals in print form and like to see their office bookshelves adorned in color-coordinated journal sets that serve as a standing monument to their academic career.

After the Second World War, publishers quickly realized the enormous profits that could be gained from this industry, and journals sprung up to represent every facet of science.

## THE LIMITATIONS OF EXISTING SYSTEMS

The inherent problems in the above system are as follows.

### Slow processing

The most vexing problem now is the unacceptable length of time that the publication process takes. The *Journal of Burns and Wounds* was born out of frustration following the enormous time delays occurring in the publication of scientific articles. Delayed publication may mean delayed promotion and delayed introduction of new ideas and innovations, and creates frustration among authors, undermining their desire to invest time and effort when their work may take many months and even years to be published. How long would it have taken to complete the Human Genome Project if each step had to have been approved by a peer-reviewed journal?

### Rising publication costs

The second problem is a pricing crisis, which is preventing access to many journals. Last year a series of “journal cost quizzes” appeared on the Internet. The results graphically demonstrated that in many instances, items such as a truck, diamond ring, or plasma television cost the same as subscriptions to a single scientific journal.

And what do the publishers get out of all this? For the 6 months ended June 30, 2006, one of the major publisher's revenues increased 11% to £1.39 billion ($2.6 billion). Their net income increased 61% to £115 million ($216 million).^1^

Faced with the fiscal realities of ever-increasing subscription costs for scientific journals, universities have begun to react to this problem. Scientists at the University of California at San Francisco urged a worldwide boycott of six molecular biology journals on the grounds that their publisher was demanding that the University of California pay $90,000 a year for electronic access to the publications. In their letter to the publisher, they noted that their university was already spending $8 million a year for online access to their journals. Other major universities have joined in this revolt and are planning to cancel hundreds of journal subscriptions over the next several years.

### Space limitations

Paper journals have a finite number of pages available in each issue. As a result, journals can only accommodate a fraction of the manuscripts submitted annually. In top-tier journals with exceptionally high submission volumes, this issue is magnified. One then must wonder if the rigorous peer-review process is indeed totally driven by scientific merit, or are spatial limitations and pragmatic editorial directives complicit in these high rates of rejection? Are the majority of papers rejected by top-tier journals truly unworthy of publication?

## THE COMING OF THE REVOLUTION

A new era has dawned whereby the fusion of new technology and development of an egalitarian system of public access has and is continuing to transform the world in which we live and with it scientific publishing. The new technology is digital electronic formatting and the system of public access is the Internet. In his book *The World Is Flat*, Thomas Friedman describes this tectonic shift and lays before the reader the realities and possibilities of the present and the future. Electronic medical publishing will certainly be a part of this new reality.

It is not widely known but physicists rarely use journals. This is because physics changes so quickly that no progress could be made employing the current paper-based system. In 1991 the US Department of Defense's Los Alamos National Laboratory created an electronic repository of technical reports for high-energy physics. Researchers deposit their work free of charge, and this information is free to readers. Authors retain the copyright, have complete control over their work, and are free to publish on the repository or in a conventional journal. This system has spread to involve the whole of physics, mathematics, and computer science.

Inspired by the above, attempts were made to create a similar biomedical electronic publishing repository at the National Institutes of Health (NIH). However, publishers and societies vigorously opposed the idea of articles being posted free on the Internet, and this E-biomed server was largely abandoned.

Undeterred by this setback, a global movement has developed calling for “open access to science.” This movement continues to grow and has as its fundamental principles the following:

- Science should have the ability to be freely available on the Internet.
- Readers should be allowed to download, print, and distribute the full text.
- Authors should retain the copyright of their work.

In 2001 the Open Society Institute (an organization backed by George Soros) met in Budapest to decide how to galvanize their resources to bring about “open access.”^2^ This was called the Open Access Initiative. Their goal was to bring about unrestricted toll-free full-text access to the entire refereed research corpus (20,000 journals, 2,000,000 articles per year) and to persuade researchers to publish papers in open access journals.

In December 2003, a United Nations World Summit on Communication Technology was held in Geneva, Switzerland. Delegates from 176 nations concluded with a commitment to universal access to all scientific knowledge. Secretary General Kofi Annan warned of the dangers of depriving the world of scientific knowledge. The plan of action called on governments to promote electronic publishing and open access initiatives, thereby making scientific information affordable and accessible in all countries on an equitable basis.

## THE SOLUTION

The National Library of Medicine's main repository remains PubMed, and the NIH recognizes its inherent limitations. In response to these shortcomings, the NIH has instituted a parallel but free digital archive of biomedical science consisting of the entire text and not merely the abstracts, named PubMed Central (PMC), that is linked to PubMed and Medline.

On July 14, 2004, the US House Appropriations Committee recommended to the NIH that it develop a policy permitting free online access (in PMC) to articles based on NIH-funded research no later than 6 months after their publication in peer-reviewed journals.^3^ Interestingly, the major publishers have opposed this recommendation.

We have joined the ranks of those who believe that access to peer-reviewed journals of the highest standard should be free to all readers and authors. The publication of scientific investigation should be in a timely manner and editorial decisions to publish or reject should occur purely on scientific merit and not the number of journal pages per month. Finally, we strongly support putting the copyright back where it belongs—in the hands of the authors.

## References

[1] Stock overview Reuters. http://today.reuters.com/stocks/overview.aspx?symbol=REL.L.

[2] Budapest Open Access Initiative http://www.earlham.edu/~peters/fos/boaifaq.htm#journals.

[3] Policy on enhancing public access to archived publications for NIH funded research http://grants.nih.gov/grants/guide/notice-files/NOT-OD-05-022.html.

